# Research Into Digital Health Intervention for Mental Health: 25-Year Retrospective on the Ethical and Legal Challenges

**DOI:** 10.2196/58939

**Published:** 2024-09-09

**Authors:** Charlotte L Hall, Aislinn D Gómez Bergin, Stefan Rennick-Egglestone

**Affiliations:** 1 Institute of Mental Health Mental Health and Clinical Neurosciences, School of Medicine University of Nottingham Nottingham United Kingdom; 2 National Institute for Health and Care Research (NIHR) Nottingham Biomedical Research Centre Institute of Mental Health University of Nottingham Nottingham United Kingdom; 3 National Institute for Health and Care Research (NIHR) MindTech HealthTech Research Centre Institute of Mental Health University of Nottingham Nottingham United Kingdom; 4 Responsible AI UK School of Computer Science University of Nottingham Nottingham United Kingdom; 5 School of Health Sciences Institute of Mental Health University of Nottingham Nottingham United Kingdom

**Keywords:** digital mental health intervention, research ethics, compliance, regulation, digital health, mobile health, mhealth, intervention, interventions, mental health, retrospective, ethical, legal, challenge, challenges

## Abstract

Digital mental health interventions are routinely integrated into mental health services internationally and can contribute to reducing the global mental health treatment gap identified by the World Health Organization. Research teams designing and delivering evaluations frequently invest substantial effort in deliberating on ethical and legal challenges around digital mental health interventions. In this article, we reflect on our own research experience with digital mental health intervention design and evaluation to identify 8 of the most critical challenges that we or others have faced, and that have ethical or legal consequences. These include: (1) harm caused by online recruitment work; (2) monitoring of intervention safety; (3) exclusion of specific demographic or clinical groups; (4) inadequate robustness of effectiveness and cost-effectiveness findings; (5) adequately conceptualizing and supporting engagement and adherence; (6) structural barriers to implementation; (7) data protection and intellectual property; and (8) regulatory ambiguity relating to digital mental health interventions that are medical devices. As we describe these challenges, we have highlighted serious consequences that can or have occurred, such as substantial delays to studies if regulations around Software as a Medical Device (SaMD) are not fully understood, or if regulations change substantially during the study lifecycle. Collectively, the challenges we have identified highlight a substantial body of required knowledge and expertise, either within the team or through access to external experts. Ensuring access to knowledge requires careful planning and adequate financial resources (for example, paying public contributors to engage in debate on critical ethical issues or paying for legal opinions on regulatory issues). Access to such resources can be planned for on a per-study basis and enabled through funding proposals. However, organizations regularly engaged in the development and evaluation of digital mental health interventions should consider creating or supporting structures such as advisory groups that can retain necessary competencies, such as in medical device regulation.

## Introduction

The World Health Organization has identified a treatment gap for mental health [1], which is present across all regions and for all mental health conditions [2]. For some conditions and in some settings, the treatment gap is increasing. For example, the depression treatment gap in the United States increased between 2015 and 2019 due to an increased depression prevalence in younger people, with no commensurate increase in treatment [3].

Digital mental health interventions (DMHIs) are now routinely integrated into mental health services internationally and can extend provision to those not currently using mental health services [4]. DMHIs can be used to support the prevention, assessment, diagnosis, treatment, and management of mental health conditions. For this review, we focus mainly on treatment and management. DMHIs can enable the delivery of treatment outside of psychiatric settings, a factor identified by a World Psychiatric Association member survey as extremely important for improving access to mental health care, particularly for health conditions other than schizophrenia and those experienced in childhood [5]. Even in the case of schizophrenia and mental health conditions experienced in childhood, studies have shown that treatment through DMHIs can be safe and effective in nonpsychiatric settings [6,7]; hence, we would argue that they are relevant transdiagnostically.

DMHIs can be more easily scalable than nondigital treatments, potentially narrowing the global treatment gap, particularly given a rapid increase in access to computing and networking technologies globally, even in low-resource settings [8]. However, despite this potential for scalability, the majority of DMHIs are not widely integrated into health care systems [9], and varying access to mobile technologies continues to be reported as a barrier to their use to narrow the treatment gap in low- and middle-income settings [10]. Furthermore, for the subset of DMHIs that integrate artificial intelligence (AI), a systematic review has highlighted biases in the mental health conditions examined in evaluations and deficits in the methodological quality of research. These examples point to continuing barriers that limit integration into evidence-based practice [11].

Over the past quarter century, the DMHI landscape has swiftly progressed, enabled by rapid technological advances during this period. This has resulted in an ever-increasing range of DMHIs, including those delivered as mobile apps, internet-delivered therapy, AI, extended and virtual reality, and remote measurement technology (to name just a few). The mode of digital complexity of these DMHIs may vary from low (eg, the digital platform used as a tool for delivery such as videoconferencing) to high (eg, personalization of therapeutic content through machine learning technologies). The level of human input into these DMHI may also vary from fully self-directed (eg, mental health app with no clinical input), to asynchronous motivational support (eg, a therapist provides motivational texts to encourage engagement with a digital tool), through to fully supported (eg, real-time delivered clinician therapy via videoconferencing) [12].

Many DMHIs are publicly available, often for free or at a low cost, such as through public app stores. Most app store offerings have no scientific support or involvement in their development process [13], with the consequence that some can recommend harmful strategies, such as consuming strong alcohol during a bipolar episode [14]. However, DMHIs have been an important focus of mental health research, including high-quality randomized controlled trials (RCTs). For example, in a recent review of trials included in the International Standard Randomized Controlled Trial Numbers (ISRCTN) registry, we identified 23 DMHI trials that had published findings [15]. Since DMHIs are delivered using digital technologies, their evaluations are frequently delivered using technology as well. In online trials, some or all trial procedures are delivered online, frequently (but not always) on the web, which brings a range of methodological challenges [16]. DMHIs often have a small effect size; a meta-analysis of evaluations of DMHIs for depression in adults found a pooled effect size of Cohen *d*=0.33 (95% CI 0.11-0.55) in evaluations of unsupervised DMHIs [17]. Since a small anticipated effect size necessitates a large target sample, just one challenge in online trials can be finding sufficient eligible participants.

The authors have published and contributed to RCTs of DMHI evaluations, including novel interventions for tics [18,19], attention deficit hyperactivity disorder (ADHD) [20], virtual reality therapy for people with psychosis [7], and the use of mental health recovery narratives as an intervention [21]. We have observed that research teams designing and delivering evaluations frequently invest substantial effort in deliberating on ethical and legal challenges around DMHIs. Conducting our own evaluations ethically and legally has been one of our primary concerns. This focus on ethics and legality can arise because evaluations of DMHIs introduce challenges that are less present in evaluations of more traditional clinical-led interventions, such as less or no contact with clinical teams and the impact this has on monitoring participants (for example, to identify adverse events). DMHIs can result in harm through different mechanisms, such as usage by people experiencing a desperate need for mental health improvement who are unsuited to a particular offering [22] but face a lower barrier to entry to a DMHI in a web-based trial with a self-rated inclusion assessment. Legal challenges can arise when there are rapid changes in international law, such as around data protection arrangements for data transfers between jurisdictions [23] and when regulatory systems are challenged by the rapidly changing landscape of DMHI provision (eg, as AI technologies are integrated) [24].

As we reflect on the past 25 years and the current position, we draw on our own research experience of DMHI design and evaluation and our knowledge of the field to identify ethical and legal concerns that we believe are of critical importance to address. We endeavor to support an informed discourse and guide future research designs to promote the ethically sound and legally resilient integration of DMHIs into mental health care provision.

## Harm Caused by Online Recruitment Work

Online evaluations of DMHI often use online recruitment mechanisms. These can enable the rapid recruitment of participants [16], including people not currently using formal health services, which is important for external validity if DMHIs are intended to extend service reach [4]. However, some forms of online recruitment can cause harm to people with mental health conditions, which means that the design of recruitment strategies is an ethical issue.

Some researchers have chosen to “lurk” on various web-based discussion forums, by joining these forums as a member solely to share recruitment material with other members. Their presence can compromise the safety of these spaces [25]. For example, author Rennick-Egglestone, who is a longstanding member of a mental health support group for people experiencing cyclothymia, has seen peers leave the group permanently when researchers join to share recruitment material, due to a fear that group interactions may be treated as data in research studies. The British Sociological Association has published general guidance on researching web-based forums, which provides a broad overview of thought and practice in this space [26]. The British Psychological Society has provided a guide to how its Code of Human Research Ethics can be interpreted in internet-mediated research [27]. Both highlight that the distinction between public and private space is increasingly blurred in web-based interactions, leading to challenging decisions for researchers about appropriate methods.

Harm can also be caused if potential participants misunderstand online recruitment material, and hence feel misled about the purpose of a study they have chosen to participate in. This may be more likely in web-based evaluations (eg, if study information such as participant information sheets is disseminated over the internet) in a context wherein study team members are not routinely available to explain its contents. We have explored the development of mandatory recruitment principles for online evaluations by developing and using exemplar principles for a web-based trial, which were intended to promote safe practices by trial workers [28]. These included explicit instructions not to lurk but instead to approach the gatekeepers of online groups with requests to share recruitment material and direction on minimum content for each published recruitment message [28].

Frequently, participants identified through web-based recruitment mechanisms are not asked for formal identity verification. This is in keeping with guidance from US [29] and UK [30] regulators on procedures for clinical trials that do not evaluate investigational medicinal products, commonly referred to as non-CTIMPs (non–Clinical Trials of Investigational Medicinal Products). There is developing evidence that “fake participation” is a risk to the integrity of studies recruiting online that do not formally confirm identity. For example, Hydock (2017) reported that a “small but nontrivial portion of participants in online survey studies misrepresented their identity for the chance of financial gain” [19]. How to identify and respond to repeat registration is an ethical consideration, as misrepresentation by participants can distort findings [31]. This could amount to a misuse of the effort contributed by real trial participants and the misuse of public funds through subsequent commissioning decisions.

Without formal identity verification, DMHIs may also be accessed by people for whom they are not suitable, which raises both legal and ethical concerns, such as if DMHIs intended for adults are accessed by children. Legally, this is a developing space, with the UK Online Safety Act (2023) and the US Children’s Online Personal Protection Act (1998) both offering children under the age of 13 greater protection in the online space (eg, restricting the data that is allowed to be collected without parental consent [32]). The latter was reinforced in the United States via the Children's Online Privacy Protection Rule introduced in 2013 [33]. Studies that inadvertently include data provided by younger children may therefore be challenged on the legality of their processes.

Some DMHIs can contain mental health material with the potential to cause distress [21], which may be unsuitable for people such as children lacking the capacity to process the distress of others. These considerations take place in a context where campaign groups across the globe have called for tighter restrictions in response to the terrible loss of children and young people to suicide attributed to the negative impact of web-based platforms [34].

## Monitoring of Intervention Safety

DMHIs can harm people with mental health conditions. The harm can arise through mechanisms such as the physical act of engaging with the intervention (eg, cybersickness in the case of virtual reality), or through interactions between the type of content or information given and the mental health experiences of the technology user. For example, in the United States, the National Eating Disorders Association was forced to remove an AI chatbot servicing a helpline when it began to provide recommendations, such as limiting caloric intake that might reasonably be expected to exacerbate conditions for people experiencing disordered eating [35]. Most generally, harm due to DMHIs might be experienced as one or more *adverse events*, some of which can be serious, including hospitalization due to substantial distress associated with intervention usage [21]. A growing body of evidence suggests that, historically, adverse events caused by DMHIs have not been well recorded either in research evaluations or in post market surveillance of DMHI usage [15]. For example, a recent review of 23 DMHI trials included in the ISRCTN registry revealed that although most (69%) have published one or more statements on adverse event identification, few (26%) had reported on the number of adverse events in their main findings [15], and few had examined whether these events were serious (26%) or related to the intervention (17%). Another review on DMHI showed that only 65% (15/23) of studies (which included trials and postmarket surveillance studies) explored risks to safety. The authors noted that this was an improvement compared with the findings of a review in 2017, whereby none of the 9 included papers assessed safety risks [36].

Despite this increase, there is a clear need to improve the way harms are monitored, both during formal research evaluations and as technologies are implemented into practice. It is critical that harms are understood to inform the benefit/risk ratio and to make informed decisions on whether a product should be implemented in health care settings, as well as on forms of support required for safe use. Recording adverse events in online evaluations brings new challenges, particularly if there are no routinely planned interactions with health care workers where adverse events could be enquired about or reported. The likelihood of adverse events being spontaneously reported is limited in online interventions, even where reporting mechanisms are provided [15]. Suggestions have been raised to facilitate harm and event identification, including ensuring independent review committees include technology experts [15], ensuring adverse events are statistically compared between arms in a trial, and using information on harms collected during an evaluation to inform postmarket surveillance [37]. There is also the potential to integrate features into DMHIs to support the recording of harms, including developing real-time monitoring of symptoms that raise alerts when participants reach predefined cut-offs. This requires evaluation to understand its acceptability, feasibility, and effectiveness [15].

## Exclusion of Specific Demographic or Clinical Groups

It is an important ethical principle that access to evidence-based health care is equitable and does not disadvantage certain groups or patient characteristics. For example, in the United Kingdom and the United States, over 80% of adults and young people own a smartphone [38-40]. However, access to a mobile data plan is likely to be less in populations that are typically underserved by research and health care services (with example populations in the United States including Black, Hispanic, older, and less educated populations) [39]; hence, evaluations and implementation of DMHIs risk exacerbating existing health inequalities [41]. Although it is anticipated that digital disparities will reduce over time, the importance of digital literacy should not be overlooked [40]. Some training projects have been developed to address issues around digital literacy, including the Digital Outreach for Obtaining Resources and Skills program, which provides both in-person and web-based support in digital literacy [42]. The use of “digital navigators,” who are trained members of the clinic team providing technology support [43], while not placing additional burden on core-delivery clinic members, is a potentially promising way forward and has been implemented successfully in DMHI evaluations [44]. It is also critical that clinicians and health care providers are efficient at integrating technology into practice to increase access to DMHI, but clinicians may worry about the capacity of clinical systems to respond to risks created by DHIs [45].

Equally, it is critical that the DMHIs are designed and evaluated in the cultural context where they will be used [46]. The majority of research on DMHI is delivered in Westernized countries with relatively high incomes; studies have suggested that the effectiveness of these interventions may not directly translate to different cultures [47,48]. For example, issues such as the concept of “depression” not translating across cultures and languages [49] highlight the critical importance of language and context [50]. Although there is a growing interest in culturally adapted DMHIs, to date there has been no research that directly compares culturally adapted versus nonadapted interventions to explore if this improves uptake and effectiveness [48]. Given the potential that DMHIs have in providing scalable health care solutions, this requires further research.

Finally, trials of DMHIs often do not include participants who may be of higher risk, such as those with suicidality or severe mental health problems. A recent review found that 13 out of 23 trials screened out patients who had more serious and enduring mental health illnesses [15]. Another review found that the main method to mitigate risk in trials of DMHIs was to exclude high-risk populations [37]. Whereas establishing proof-of-concept and indications of efficacy and safety on a restricted sample is ethically appropriate, it is important that when larger, pragmatic trials or research designs are conducted before wide-scale rollout, these larger studies should include samples that are reflective of the population of interest, including all relevant characteristics and comorbidities. The severity of mental health problems is frequently underdiagnosed; hence, evaluations should consider that DMHIs may be used by people whose mental health conditions are much more severe than for whom the intervention was intended, such as people diagnosed with mild-to-moderate depression, but who are experiencing a period of severe depression [22].

Some categories of DMHI may embed a risk of discrimination on personal characteristics that are protected by law. One example is AI technologies, where evidence shows that algorithmic biases can lead to discrimination based on characteristics such as gender and ethnicity [51], which are frequently protected in national legislation [52]. Laws provide protection for individuals against discrimination, but the challenge is often understanding when discrimination has happened, as the algorithms used in interventions often can be difficult to interpret and explain [51].

## Inadequate Robustness of Effectiveness and Cost-Effectiveness Findings

The adequacy of study designs to fully assess intervention effectiveness and cost-effectiveness is a key challenge to the field. If trial designs are not sufficiently robust to answer the intended question, it is possible that the reported benefits of an intervention may be inflated, resulting in interventions that are not effective or fit-for-purpose being recommended for routine use. Consequently, engaging in these interventions may result in users delaying access or being unable to access more suitable interventions, and potentially represents a waste of health service resources, hence the adequacy of evaluation design is inherently an ethical issue.

One significant challenge lies in selecting the most appropriate comparator for RCTs. Potential comparators include active therapeutic interventions delivered nondigitally (eg, face-to-face therapy) or different active interventions (eg, cognitive-behavioral therapy versus exposure therapy) delivered digitally. Alternatively, comparators may be nonactive controls such as waitlists or no intervention. It may be impossible for participants to be masked to treatment allocation in a DMHI evaluation, raising the risk of bias. Reviews have suggested that comparisons to nonactive controls may lead to larger treatment effects than comparisons to active controls [53,54].

Trials of DMHI frequently encounter high dropout rates (failure to follow participants up to the primary and secondary end points) or fail to recruit the target number of participants, leading to reduced statistical power and a lower probability of identifying effects. Analysis of data from the National Institute for Health and Care Research (NIHR) Health Technology Assessment database revealed that from 2010 to 2020, 60% of DMHI trials did not meet their recruitment targets, and 56% did not meet their follow-up targets [55]. This shortfall may result in recommendations based on insufficient research to draw definitive conclusions. It raises an ethical concern about wasting the time of participants who have engaged in the research but to no scientific avail.

A significant challenge within DMHIs is the speed at which technology evolves and progresses, versus the slow and careful study designs we use, predicated on pharmaceutical research. By the time RCTs are complete, the technology itself may have required significant changes to be made within the intervention. This raises the question as to whether we should be considering alternative study designs (eg, rapid evaluations of specific therapeutic components or evaluating the application of therapeutic modalities through different mediums rather than specific interventions).

A systematic review has shown that current study designs often neglect to assess the cost-effectiveness of interventions [56]. Understanding cost-effectiveness is crucial for evaluating an intervention's benefits to the health care system and enabling informed decisions by health care providers regarding investment in digital technology over other treatment modalities. Moreover, if trials do not explore cost-effectiveness, they may fail to record other health resource utilization outside the intervention, which is essential for understanding clinical benefits between treatment groups.

## Adequately Conceptualizing and Supporting Engagement and Adherence

Most DMHIs require some form of active engagement from their users. Some interventions, such as Beating the Blues [57], are built on an interaction approach in which users are expected to work through a structured program of activities intended to offer therapeutic benefits, often intended to last for weeks or months. Developing DMHIs with the potential to sustain engagement is a significant challenge; research studies and real-world implementation experiences often report low levels of engagement [55]. Where engagement is low or irregular, and engagement with a structured program of activities is intended, this is sometimes conceptualized by study teams as poor adherence to the intended technology interaction. Poor adherence to structured programs is frequently reported in research evaluations [55].

Challenges around engagement and adherence are ethical issues for research teams. For example, an automated DMHI may present a user experience that is off-putting due to poor design. This might cause harm if a person with a mental health condition perceives it as a personal failing that they could not sustain the expected engagement, rather than a technology design failure. Whether to include human support in study designs is an important question. Studies have consistently demonstrated that guided interventions, which involve human support through asynchronous messages or real-time interaction, have demonstrated higher adherence and greater clinical effectiveness compared to unguided interventions [58], but there are settings in which they may not be practical (eg, due to resource limitations) or where they may cause harm, such as where the expected user base is experiencing profound social anxieties [22].

Engagement with a DMHI is often difficult for researchers to quantify. Often, a range of DMHI interaction behaviors may need to be considered, including frequency or duration of interaction with the DMHI, and metrics of “active” engagement,” such as the number of clicks on links, pages visited, or interactive tasks completed [59]. It can be difficult for researchers to define which is the best metric to use. One approach is to conduct a principal component analysis to create a comprehensive composite measure [59]. Some researchers are beginning to explore more subjective constructs of engagement, including aspects such as knowing that the intervention is available whenever it is needed, and satisfaction and usability [60].

Despite its importance, research evaluations of internet-delivered interventions often neglect to investigate the impact of usage level on intervention outcome. A systematic review found that only 21% of included trials had conducted statistical analyses to account for differences in usage [61], leading to a lack of knowledge on how much engagement with a DMHI is sufficient. Even if an understanding of the necessary level of engagement to provide benefits is established in a tightly controlled research setting, achieving this in a real-world setting can be challenging, as research settings often offer greater levels of participant support than in real-world implementation. For example, when the serious game “SPARX” aimed at supporting low mood in children and young people was nationally rolled out in New Zealand, only between 2% and 5% of young people completed the full program [62], whereas considerably higher completion rates had been found in the trial [63].

There is also a danger of research teams misconceptualizing lack of usage as poor adherence, in cases where users have made a sensible choice to avoid usage of a technology that provides no benefits or is actively harming them. This can occur if teams take an ego-centric approach to conceptualizing their offerings as of central importance to their users [64]. For a recent trial examining the benefits of receiving access to a collection of mental health recovery narratives [21], we chose not to require a minimum level of usage, as our empirically developed change model had established that access to narratives could cause harm, so we wanted to enable users to self-manage engagement, which could include no engagement [65,66]. Healthy engagement was supported by a range of safety strategies, including the use of content warnings [66]. Hence, in this example, we did not conceptualize low engagement as a lack of adherence [21].

## Structural Barriers to Implementation

DMHIs often struggle to bridge the gap from a research product to implementation in health care services or broader society [67]. This is an ethical issue, as research evaluations regularly require hundreds of participants, large teams of evaluators and support workers, and access to substantial amounts of public funds, all of which are underused if this transition is not made. If DMHIs are both clinically effective and cost-effective but not implemented, people with mental health problems may not be receiving optimal care. Currently, there are several DMHIs that are available for use in health service settings that have not undergone independent evaluation [9]. Conversely, there are several well-validated DMHIs that may never be implemented in routine health care settings [68]. This illustrates a possible disconnect between research-led efforts to evaluate interventions, and the needs of clinical practice, which is a major barrier to service user benefit.

There is likely to be a broad range of factors underpinning this disconnect, and addressing these is critical to reducing the underuse of research evaluations of DMHIs. The fast-paced nature of technological progress can mean DMHIs can become obsolete or unacceptable to users before they reach the implementation stage [53] due to the slow progress of the research lifecycle. Research teams may lack the institutional support or commercial knowledge needed to implement DMHIs that they have evaluated, and work to implement technologies may be at odds with funder or institutional metrics used to evaluate individual academic performance, particularly where the regulatory burden is high. A possible solution is government-funded infrastructure to support the integration of academic, clinical, and industry expertise, but substantial resources may be required.

A clinically grounded challenge is that health care professionals may not have received preregistration training or continuous professional development in DMHIs and how to work with them clinically, and hence may lack the competence or confidence to integrate them into care. For example, an interview study with Norwegian general practitioners interested in computerized cognitive behavioral therapy revealed their concerns that they had insufficient knowledge to effectively recommend these to their service users [69]. We have argued that health care practitioners need training to enable their own psychological safety when providing therapies online through videoconferencing services, including due to the lesser availability of immediate emergency response in the event of suicidality [70].

## Data Protection and Intellectual Property

The deployment and evaluation of DMHIs typically involve processing personal and sensitive data, sometimes with processing through third parties such as web-server hosts. In many jurisdictions, these data will be protected by legislation. For example, in the European Union (EU), Article 8 of the European Convention on Human Rights and Articles 7 and 8 of the EU Charter of Fundamental Rights enshrine privacy as a fundamental human right, and national implementations of the General Data Protection Regulations provide a specific legal framework for data processing.

Web-based DMHIs, in particular, have the potential to be evaluated in one jurisdiction but incorporate components hosted in another, frequently requiring close attention to the navigation of legal requirements, especially when the countries involved have different standards of data protection [71]. Additional challenges are present when computer vision technologies such as augmented reality are used, as data about third parties may be collected without their consent. Service users and clinicians have raised concerns about data security in DMHIs and the potential for unauthorized breaches of sensitive data [71-73]. For health care services, the introduction of these technologies may present too much uncertainty in terms of their legal requirements. This can contribute to challenges with the implementation of DMHIs. If users are encouraged to create and share content as part of working with a DMHI, this introduces additional considerations around how such content is licensed and used [74].

As evaluators, we have all invested substantial effort in data management planning around DMHIs, including identifying how to ethically and legally work with the data that is generated through our evaluations. This requires access to specific competencies such as knowledge of data protection laws. These competencies require work to maintain, as regulations around data processing can change rapidly (eg, when the EU Safe Harbor scheme for cross-border data processing was replaced by Privacy Shield [23]). Legal and ethical issues are present even for data that is publicly available. In 2015, the Samaritans released an app that identified tweets associated with suicidal ideation and alerted friends of those tweeting. This was rapidly withdrawn following interventions by privacy campaigners [75]. The UK Information Commissioner’s Office subsequently found that it was unlikely to be compatible with UK data protection law [76]. More recently, the US Federal Trade Commission fined BetterHelp, a platform connecting people to therapists, $7.8 million for sharing IP and email addresses as well as sensitive mental health information to third parties [77].

There are similar challenges around intellectual property, as DMHIs may emerge from collaborative efforts involving multiple partners, and they may be built on underlying technologies such as software libraries produced by third parties, sometimes with restrictive licensing conditions that can preclude commercial use. Successfully addressing these issues requires a proactive stance from the outset of development. We advocate seeking guidance from legal or technology transfer teams early in the conceptual phase to mitigate risks. Moreover, there is a pressing need to enhance researchers' skills in translational research and foster collaborative endeavors between stakeholders to effectively support the future implementation of DMHI.

## Regulatory Ambiguity Relating to DMHIs That Are Medical Devices

Several organizations across the globe regulate hardware and software artifacts classified as medical devices. These include the European Medicines Authority, the Medicines and Health Care Products Regulatory Agency (MHRA) in the United Kingdom, the Food and Drug Administration (FDA) in the United States, and the Therapeutic Goods Administration (TGA) in Australia. These organizations typically have legislative powers to ensure that medical devices are safe and effective, which can include requiring manufacturers to submit information on their approach to the safe storage and processing of data. How this is enacted and how medical devices are defined may differ, and some countries remain without regulatory authorities. Where medical devices are regulated, classification is usually based on the “intended use/purpose,” as defined by the manufacturer or developer. For example, the EU’s classifications for medical devices are outlined in Textbox 1 [78].

**European Union (EU) classifications for medical devices.**

“Medical device” means any instrument, apparatus, appliance, software, implant, reagent, material, or other article intended by the manufacturer to be used, alone or in combination, for human beings for one or more of the following specific medical purposes:

- diagnosis, prevention, monitoring, prediction, prognosis, treatment, or alleviation of disease;
- diagnosis, monitoring, treatment, alleviation of, or compensation for, an injury or disability,
- investigation, replacement, or modification of the anatomy or of a physiological or pathological process or state;
- providing information by means of in vitro examination of specimens derived from the human body, including organ, blood, and tissue donations;
- and which does not achieve its principal intended action by pharmacological, immunological, or metabolic means, in or on the human body, but which may be assisted in its function by such means.

This definition explicitly recognizes the concept of Software as a Medical Device (SaMD), which is of particular relevance to DMHI developers and evaluators. The use of terms such as “disease” and “disability” have often left medical device classification ambiguous for SaMD intended for digital mental health purposes. In some jurisdictions, amendments have been made to reduce ambiguity. For example, the FDA has separated out a category of “low-risk devices” intended to target wellness or well-being, by offering specific internal staff guidance on how they should be regulated [79]. This perhaps creates a risk of DMHI developers classifying their tools as “wellness” devices rather than “treatments” to reduce regulatory burden [40]. In the United Kingdom, the MHRA is engaged in a change program on SaMD regulation but with a component examining arrangements needed so that AI technologies are effectively regulated [24].

Resolving ambiguity around whether our interventions are medical devices has sometimes taken substantial effort for us as evaluators (eg, through seeking opinions from sponsors and legal experts), and we are aware of at least one major research program that faced a delay of over a year when its intervention was unexpectedly classified as a medical device. We also wonder whether DMHIs are underclassified as medical devices; our review of DMHI trials found that only 2 of 23 interventions had a medical device classification [15]. Given the ambiguity of regulation, there is a potential for the medical status of DMHIs to change between the awarding of funding and the start of evaluation. We have found that regulatory guidance can be more ambiguous where DMHIs are not classified as medical devices, perhaps due to a regulatory perception that harm is less likely. For these DMHIs, authors ADGB and CLH are working to produce adverse event reporting guidelines that complement those of the MHRA.

Regulations that define when a medical device needs to be clinically investigated can also create ambiguity for DMHIs. For example, in MHRA regulation, clinical investigation is not needed if there is sufficient evidence available for an intervention that is equivalent [80]. Equivalency might be straightforward to assess for medication but less clear for DMHIs (eg, does equivalency hold if the same therapeutic intervention is delivered but through a different device?). Within MHRA regulation, further clinical investigation is needed when there has been a significant change to an intervention. However, current regulations may be unclear for AI-based DMHI interventions that learn and hence evolve; therefore, regulation is currently being examined in this space [24]. Finally, DMHIs are sometimes used outside their intended purpose (eg, if a self-management intervention is evaluated to treat a condition such as depression). These “secondary uses” for digital technologies appear to be relatively common and may produce harms that are not recorded (eg, if participants drop out of an evaluation due to being given an inappropriate technology and are not followed up).

## Conclusion

Over the past 25 years, the rapid evolution of both DMHI-based research and encompassing regulatory systems has meant that study teams are faced with multifaceted ethical and legal challenges to resolve. We have drawn on our own experiences to describe 8 of the most pertinent challenges, but there will be others, and some concerns will specifically apply to particular types or forms of DMHI. Collectively, the challenges we have identified highlight a substantial body of knowledge and expertise that is required on the part of study teams, either within the team or through access to external experts. The challenges that we present also illustrate potentially serious negative consequences if sufficient resources are not available to study teams, including challenges regarding the legality of data collection procedures that inadvertently collect data from children, harm caused to members of the public through unethical recruitment methods, and substantial delays to studies that discover medical devices legislation applies to their intervention. Our focus was predominately on treatment; however, a similar reflection of the challenges of DMHI for prevention, assessment, or diagnosis is also warranted.

In many cases, understanding how to respond to these challenges requires collaboration between industry representatives, academics, public contributors, and clinicians. Each of these stakeholder groups can bring unique expertise and perspectives, and by leveraging their collective insights, innovative solutions can be developed to promote the legal and ethical use of DMHIs. To support success, such collaborations need to be planned early, with relevant collaborators and financial resources integrated into funding proposals, or other mechanisms for collecting study resources. For example, resources to pay for legal opinions are frequently not included in proposals but may be necessary to support successful negotiation of regulatory processes.

Organizations conducting health research might consider how to create structures that develop and maintain the necessary competence to resolve the challenges that we have identified. For example, organizations that are frequently engaged in DMHI evaluation might consider developing specific competence on medical device regulations, including the capacity to conduct horizon-scanning activities to identify possible changes (given that regulations can change between proposal submission and evaluation conclusion). Organizations might also consider developing communities of public members interested in public involvement in research. Collectively, we have found discussions with public members essential to resolve the most challenging ethical issues, such as around the management of narrator identity in our trial of a DMHI integrating mental health recovery narratives [66].

## Abbreviations

ADHD: attention deficit hyperactivity disorder
AI: artificial intelligence
CTIMP: Clinical Trial of an Investigational Medicinal Product
DMHI: digital mental health intervention
EU: European Union
FDA: Food and Drug Administration
ISRCTN: International Standard Randomized Controlled Trial Number
MHRA: Medicines and Healthcare Products Regulatory Agency
NIHR: National Institute for Health and Care Research
RCT: randomized controlled trial
SaMD: Software as a Medical Device
TGA: Therapeutic Goods Administration
